# Biochemical and X-ray analyses of the players involved in the *faRel*2/*aTfaRel*2 toxin–antitoxin operon

**DOI:** 10.1107/S2053230X23007288

**Published:** 2023-09-20

**Authors:** Lucia Dominguez-Molina, Ariel Talavera, Albinas Cepauskas, Tatsuaki Kurata, Dannele Echemendia-Blanco, Vasili Hauryliuk, Abel Garcia-Pino

**Affiliations:** a Cellular and Molecular Microbiology, Faculté des Sciences, Université Libre de Bruxelles (ULB), Boulevard du Triomphe, Building BC (1C4 203), 1050 Brussels, Belgium; b Department of Experimental Medical Science, Lund University, Lund, Sweden; c University of Tartu Institute of Technology, Tartu, Estonia; d Science for Life Laboratory, Lund, Sweden; Sungkyunkwan University School of Medicine, Republic of Korea

**Keywords:** ATfaRel2–FaRel2 complex, *Coprobacillus*, toxin–antitoxin modules, alarmones, toxSAS, RSH proteins, tRNA modification

## Abstract

The *aTfaRel*2/*faRel*2 operon from *Coprobacillus* sp. D7 encodes a bicistronic type II toxin–antitoxin (TA) module. The FaRel2 toxin is a toxic small alarmone synthetase (toxSAS) that inhibits translation through the pyrophosphorylation of uncharged tRNAs at the 3′-CCA end. The toxin is neutralized by the antitoxin ATfaRel2 through the formation of an inactive TA complex. Here, the production, biophysical analysis and crystallization of ATfaRel2 and FaRel2 as well as of the ATfaRel2–FaRel2 complex are reported. ATfaRel2 is monomeric in solution. The antitoxin crystallized in space group *P*2_1_2_1_2 with unit-cell parameters *a* = 53.3, *b* = 34.2, *c* = 37.6 Å, and the best crystal diffracted to a resolution of 1.24 Å. Crystals of FaRel2 in complex with APCPP, a non­hydrolysable ATP analogue, belonged to space group *P*2_1_, with unit-cell parameters *a* = 31.5, *b* = 60.6, *c* = 177.2 Å, β = 90.6°, and diffracted to 2.6 Å resolution. The ATfaRel2–FaRel2^Y128F^ complex forms a heterotetramer in solution composed of two toxins and two antitoxins. This complex crystallized in two space groups: *F*4_1_32, with unit-cell parameters *a* = *b* = *c* = 227.1 Å, and *P*2_1_2_1_2_1_, with unit-cell parameters *a* = 51.7, *b* = 106.2, *c* = 135.1 Å. The crystals diffracted to 1.98 and 2.1 Å resolution, respectively.

## Abstract 2

Crystallization strategies for and structural analyses of all of the players in the *faRel*2/*aTfaRel*2 toxin–antitoxin system are reported.

## 1. Introduction

Bacterial toxin–antitoxin (TA) systems are encoded by (typically) bicistronic bacterial operons and contribute to phage defence and stress resistance as well as to the stabilization of plasmids and genomic islands (LeRoux & Laub, 2022 ▸; Zhang *et al.*, 2022 ▸). Based on their nature (protein or RNA) and their mode of inhibition, TA systems have been classified into eight types (Jurėnas *et al.*, 2022 ▸; Page & Peti, 2016 ▸). The best characterized TA modules belong to type II, where both components are proteins and the antitoxin neutralizes the toxin by direct interaction. TA toxins target essential cellular processes, replication, transcription and translation, commonly causing irreversible modification of the target and rapid growth arrest (Harms *et al.*, 2018 ▸; Jurėnas *et al.*, 2017 ▸, 2022 ▸). While toxin neutralization can be achieved via different mechanisms, in type II TA systems inhibition commonly occurs by the formation of stable toxin–antitoxin complexes, which are often engaged co-translationally via an intrinsically disordered region (IDR) of the antitoxin (Loris & Garcia-Pino, 2014 ▸; Page & Peti, 2016 ▸).

The expression of TA systems is often tightly autoregulated, with the antitoxin binding to inverted repeats in the promoter region to inhibit transcription of the operon (Garcia-Pino *et al.*, 2016 ▸; Talavera *et al.*, 2019 ▸). This interaction is mediated by the dedicated DNA-binding domain of the antitoxin (Loris & Garcia-Pino, 2014 ▸). In this dual functionality of the antitoxins underlies the extreme promiscuity of TA modules observed across genomes, with both functional modules (the DNA-binding domain and the toxin-neutralization element) being exchangeable across different TA families (Jurėnas *et al.*, 2022 ▸; Loris & Garcia-Pino, 2014 ▸).

The high degree of horizontal mobility of TA operons combined with the sheer diversity of neutralization mechanisms, often involving unstructured domains, fuels the high rates of evolution leading to the diversification of TA operons (Aakre *et al.*, 2015 ▸). Many TA toxins are evolutionarily related to bacterial housekeeping enzymes. Notable examples include toxins from the Fic/Doc family (Garcia-Pino *et al.*, 2008 ▸, 2014 ▸), toxic small alarmone synthetases (toxSAS), which are members of the RelA–SpoT homolog (RSH) protein family (Brown *et al.*, 2016 ▸; Steinchen *et al.*, 2015 ▸; Tamman *et al.*, 2023 ▸; Zhang *et al.*, 2022 ▸), as well as bacterial acetyltransferases (Jurėnas *et al.*, 2017 ▸, 2019 ▸). toxSAS serve as bacterial stress response factors that produce a (pp)pGpp alarmone (Nanamiya *et al.*, 2008 ▸; Geiger *et al.*, 2014 ▸). CapRel^SJ46^, a fused toxSAS TA, has recently been demonstrated to mediate bacterial defence against phages via abortive infection (Zhang *et al.*, 2022 ▸). Once activated, CapRel^SJ46^ (and the majority of known toxSAS) corrupts protein synthesis by pyrophosphorylating the 3′-OH of the adenine residue of 3′-CCA uncharged tRNA (Kurata *et al.*, 2021 ▸). Other toxSAS synthesize the (pp)pApp alarmone, which results in depletion of the cellular ATP pool (Jimmy *et al.*, 2020 ▸). Both groups of TA toxins can be neutralized directly by type II antitoxins or by small alarmone hydrolase (SAH) antitoxins that remove the pyrophosphate group (Jimmy *et al.*, 2020 ▸).

The core of these enzymes is conserved and consists of an arrangement of β-strands and α-helices that fold together to form the active pocket. This results in a twisted β-sheet surrounded by α-helices, where the catalytic residues are situated to ensure the transfer of the pyrophosphate group of ATP to the corresponding substrate ATP, GDP, GTP or tRNA (Ahmad *et al.*, 2019 ▸; Steinchen *et al.*, 2015 ▸; Tamman *et al.*, 2020 ▸; Zhang *et al.*, 2022 ▸).

The regulation of these toxSAS has only been structurally characterized for Tas1 (Ahmad *et al.*, 2019 ▸) and CapRel ^SJ46^. Tas1 [type VI secretion effector (p)ppApp synthetase 1] catalyses the synthesis of (pp)pApp, which leads to ATP depletion in the targeted cell. The immunity factor Tis1 binds to Tas1 and hinders its activity by obstructing access of the acceptor ATP nucleotide (Ahmad *et al.*, 2019 ▸). In contrast, CapRel^SJ46^ features an autoinhibitory domain that, unlike Tis1, hinders the binding of the pyrophosphate donor ATP (Zhang *et al.*, 2022 ▸).

FaRel2 from *Coprobacillus* sp. D7 uses ATP to pyrophosphorylate the 3′-CCA end of uncharged tRNA (Kurata *et al.*, 2021 ▸). Its toxicity is counteracted by its type II cognate antitoxin ATfaRel2 via the formation of a tight complex (Kurata *et al.*, 2021 ▸). In this work, we describe the purification, crystallization and X-ray diffraction experiments of the antitoxin ATfaRel2, FaRel2–ATfaRel2 and the complex of FaRel2 with the ATP analogue APCPP. We expect that the resulting structures will shed light on the molecular basis of the toxicity and regulation mechanisms of the FaRel2–ATfaRel2 system and reveal which residues of FaRel2 are involved in the binding and hydrolysis of ATP. Furthermore, the structure of the FaRel2–ATfaRel2 complex will contribute to elucidating the mechanism of inhibition of the toxic effect of FaRel2.

## 2. Materials and methods

### 2.1. Protein expression and purification

#### 2.1.1. Antitoxin ATfaRel2

ATfaRel2 was expressed from pET-24d_His1a-HisTEV-*Coprobacillus* sp. (VHp364), where the ATfaRel2 antitoxin has a 6×His tag followed by a TEV protease recognition site. Cultures were grown in LB medium supplemented with kanamycin (50 µg ml^−1^) at 37°C with aeration. Expression was induced with 0.5 m*M* isopropyl β-d-1-thiogalactopyranoside (IPTG) when the cells carrying the plasmid reached an OD_600 nm_ of ∼0.5–0.8. The cells were harvested 16 h after induction by centrifugation and resuspended in buffer (25 m*M* HEPES pH 7.6, 1 *M* NaCl, 5 m*M* MgCl_2_, 1 m*M* TCEP, 0.0002% mellitic acid) supplied with cOmplete Protease-Inhibitor Cocktail (Roche). The resuspended cells were flash-frozen in liquid nitrogen and stored at −80°C prior to further applications.

The cell extracts were lysed using an Emulsiflex cell disruptor and the lysate was centrifuged to remove cell debris for 45 min at 25 000*g*. The supernatant was loaded onto a 1 ml HiTrap Ni–NTA column (Cytiva) coupled to an FPLC (ÄKTAexplorer) equilibrated with buffer *A* (25 m*M* HEPES pH 7.6, 1 *M* NaCl, 5 m*M* MgCl_2_, 1 m*M* TCEP, 0.0002% mellitic acid, 20 m*M* imidazole). The column was washed with a linear gradient of buffer *B* (25 m*M* HEPES pH 7.6, 1 *M* NaCl, 5 m*M* MgCl_2_, 1 m*M* TCEP, 0.0002% mellitic acid, 500 m*M* imidazole). The fractions containing His_6_-TEV-ATfaRel2 were concentrated using 3 kDa spin filters (Amicon) and loaded onto a Superdex 75 Increase 10/30 size-exclusion chromatography (SEC) column (Cytiva) equilibrated with 25 m*M* HEPES pH 7.6, 200 m*M* NaCl, 2 m*M* MgCl_2_, 0. 0002% mellitic acid, 1 m*M* TCEP. The purity of the protein was analysed by SDS–PAGE.

#### 2.1.2. Generation of catalytically impaired FaRel2^Y128F^ for structural biology

To counter the intrinsic toxicity of FaRel2, we generated a catalytically impaired version of the toxin with a Y128F substitution in the G-loop (FaRel2^Y128F^). The mutation in *faRel*2 was initially introduced into the pBAD33-FaRel2 toxSAS plasmid (Jimmy *et al.*, 2020 ▸) using the primers F-BmtI SAS Copro (GATCgctagcATGTACATCCTGGATAAGAT) and R-SAS Copro (gccgaagctTAAATTTTCTTGCAGTG). The PCR product was treated with DpnI to remove the template plasmid, purified on a PCR purification column (Sigma), phosphorylated and ligated by T4 ligase. The ligation mixture was transformed into *Escherichia coli* strain MC1061 by electroporation. The sequence of the resulting pBAD33-FaRel2Y128F_toxSAS plasmid was confirmed by sequencing (Eurofins Genomics).

The *faRel*2^Y128F^ gene was amplified from pBAD33-FaRel2Y128F using Q5 High-Fidelity Polymerase and treated with DpnI. The *faRel*2^Y128F^ gene was inserted into a modified pET-28b vector by restriction cloning with the enzymes BmtI and HindIII. The modified pET-28b already contained a six-His tag (6×His) as well as a SUMO cleavage site. The sequence of pET-28b-6×His-SUMO-*faRel*2^Y128F^ (Table 1 ▸) was confirmed by sequencing (Genewiz).

#### 2.1.3. Preparation of FaRel2^Y128F^

Cultures of *E. coli* strain BL21(DE3) transformed with pET-28b-6×His-SUMO-*faRel*2^Y128F^ were grown in LB medium supplemented with kanamycin (50 µg ml^−1^) at 37°C with aeration. When the cell culture reached an OD_600 nm_ of ∼0.5–0.8, expression was induced with 0.5 m*M* IPTG. After 3 h of induction, the cells were harvested by centrifugation and resuspended in buffer (100 m*M* K_2_HPO_4_/KH_2_PO_4_ pH 8.0, 500 m*M* NaCl, 500 m*M* KCl, 1 m*M* TCEP, 1% glycerol, 20 m*M* imidazole, 0.0002% mellitic acid) supplied with cOmplete Protease-Inhibitor Cocktail (Roche). The cells were flash-frozen in liquid nitrogen and stored at –80°C prior to further applications.

The purification was carried out similarly to that of ATfaRel2, except for the buffers: buffer *A* consisted of 100 m*M* K_2_HPO_4_/KH_2_PO_4_ pH 8.0, 500 m*M* NaCl, 500 m*M* KCl, 1 m*M* TCEP, 20 m*M* imidazole and buffer *B* consisted of 100 m*M* K_2_HPO_4_/KH_2_PO_4_ pH 8.0, 500 m*M* NaCl, 500 m*M* KCl, 1 m*M* TCEP, 500 m*M* imidazole. The fractions containing His_6_-SUMO-FaRel2 were pooled together, concentrated and loaded onto a Superdex 75 Increase 10/30 SEC column (Cytiva) equilibrated in buffer GF (25 m*M* HEPES pH 7.6, 1 *M* NaCl, 2 m*M* MgCl_2_, 1 m*M* TCEP).

The His_6_-SUMO tag was cleaved from His_6_-SUMO-FaRel2^Y128F^ by adding UlpI protease in a 1:100 molar ratio and incubating overnight at 4°C. To separate FaRel2^Y128F^ from UlpI protease and free His_6_-SUMO, the mixture was applied onto a gravity-flow TALON column previously equilibrated with buffer GF. FaRel2^Y128F^ was recovered after washing the column with buffer *C* (25 m*M* HEPES pH 7.6, 1 *M* NaCl, 2 m*M* MgCl_2_, 1 m*M* TCEP, 20 m*M* imidazole) for elution. The quality of cleavage was then analysed by SDS–PAGE and complete removal of the tag was confirmed by a Western blot using an anti-6×His-Tag Monoclonal Antibody (Thermo Fisher, catalogue No. MA1-21315-D680). The elution fractions containing FaRel2^Y128F^ were then concentrated using 10 kDa cutoff spin filters and loaded onto a Superdex 75 10/30 SEC column (Cytiva) equilibrated with buffer GF.

#### 2.1.4. Preparation of the ATfaRel2–FaRel2^Y128F^ complex

The ATfaRel2–FaRel2^Y128F^ complex was prepared by adding ATfaRel2 to FaRel2^Y128F^ in a 1.2:1 molar ratio. The mixture was injected onto a Superdex 75 16/60 column (Cytiva) and the peak corresponding to the ATfaRel2^Y128F^–FaRel2^Y128F^ complex was collected and analysed by SDS–PAGE.

### 2.2. Crystallization

Crystallization conditions were screened at 20 and 4°C by the sitting-drop vapour-diffusion method. The drops were set up in Swissci (MRC) 96-well 2-drop UVP sitting-drop plates. The drops consisted of 0.1 µl protein solution plus 0.1 µl reservoir solution and were equilibrated against 80 µl reservoir solution. Crystallization conditions were tested with several commercially available screens: Crystal Screen, Crystal Screen 2 (Hampton Research), HELIX, PACT *premier* (Molecular Dimensions), LMB Crystallization Screen (Molecular Dimensions), SG1 (Molecular Dimensions) and NeXtal (Qiagen). The protein concentration was determined from the absorbance at 280 nm and was corrected using the theoretical extinction coefficients estimated by *ProtParam* (Gasteiger *et al.*, 2003 ▸).

For the co-crystallization of FaRel2^Y128F^ with the ATP analogue APCPP (Jena Bioscience catalogue No. NU-421), fresh FaRel2^Y128F^ concentrated to 19 mg ml^−1^ was mixed with APCPP at 100 m*M* and incubated for 10 min at room temperature. ATfaRel2 was used at 12 mg ml^−1^, while the ATfaRel2–FaRel2Y^128 F^ complex was concentrated to 10 mg ml^−1^ for crystallization.

### 2.3. X-ray data collection and analysis

Prior to data collection, the crystals were transferred to a suitable cryoprotectant solution (Table 2 ▸) and flash-cooled in liquid nitrogen. Data were collected on the PROXIMA-1 and PROXIMA-2 beamlines at the SOLEIL synchrotron facility, Gif-sur-Yvette, France (ATfaRel2 and the FaRel2^Y128F^–APCPP and ATfaRel2–FaRel2^Y128F^ complexes) and at the Diamond Light Source (DLS) synchrotron, Oxfordshire, UK (ATfaRel2–FaRel2^Y128F^ complex) and were recorded on EIGER detectors. All data sets were indexed, integrated and scaled using *autoPROC* (Vonrhein *et al.*, 2011 ▸). *AutoPROC* also makes use of the programs *XDS* (Kabsch, 2010 ▸), *POINTLESS* (Evans, 2007 ▸), *AIMLESS* (Evans & Murshudov, 2013 ▸) and others from the *CCP*4 suite (Agirre *et al.*, 2023 ▸).

### 2.4. Isothermal titration calorimetry

The 150 bp DNA fragment used for ITC is listed in Table 3 ▸. The duplex was reconstituted from synthetic single-stranded oligonucleotides (Sigma) that were annealed by heating to 85 C° and slowly cooling to room temperature.

The ITC measurements were carried out at 20°C in an Affinity ITC calorimeter (TA Instruments). Prior to the experiments, ATfaRel2 and the 150 bp operator fragment were dialyzed, in the same reservoir, against 25 m*M* HEPES pH 7.6, 300 m*M* NaCl, 2 m*M* MgCl_2_, 1 m*M* TCEP. The concentration of ATfaRel2 in the 177 µl cell was 10 µ*M* and the 2 µl injections contained 115 µ*M* of the *faRel*2/*aTfaRel*2 DNA operator. The complete data processing and analysis was performed with *NanoAnalyze* (TA Instruments).

### 2.5. *In vivo* toxicity neutralization assays

Three vectors were generated for this study. Two pBAD33 vectors with arabinose-inducible promoters were generated: one encoding FaRel2 (designated pBAD33_FaRel2) and the other encoding the FaRel2^Y128F^ mutant (designated pBAD33_FaRel2^Y128F^). Additionally, a pKKD vector with an IPTG-inducible promoter was created to express ATfaRel2 (designated pKKD_ATfaRel2).

*E. coli DJ624Δara* cells were subjected to individual transformations with each of the vectors. Moreover, co-transformations were performed by introducing pKKD_ATfaRel2 along with either pBAD33_FaRel2 or pBAD33_FaRel2^Y128F^ into the cells. pBAD33 vectors provide resistance to chloramphenicol, while pKKD vectors provide resistance to ampicillin.

The cell cultures were grown overnight at 37°C in liquid LB medium supplemented with the appropriate antibiotics. Cultures were diluted serially (tenfold) and 10 µl of each dilution were spotted into LB agar plates supplied with the required antibiotics and either 1% glucose (repressing conditions) or 0.2% arabinose plus 1 m*M* IPTG (inducing conditions). Plates were scored after overnight incubation at 37°C.

## 3. Results

### 3.1. Production of ATfaRel2, FaRel2^Y128F^ and the ATfaRel2–FaRel2^Y128F^ complex

**Figure 1:**
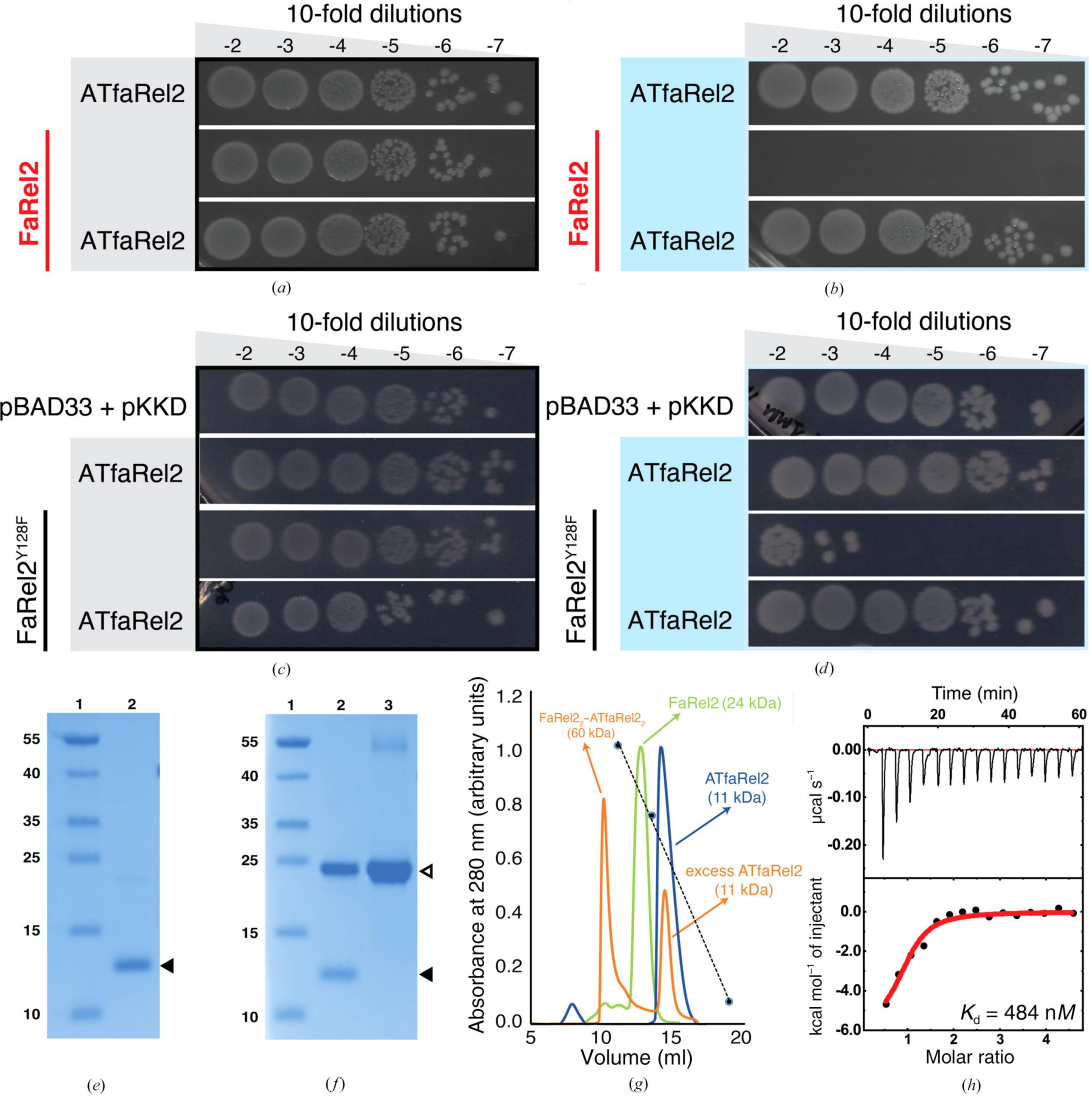
Microbiological and biochemical characterization of the proteins from the *faRel*2/*aTfaRel*2 TA operon. (*a*–*d*) *In vivo* toxicity neutralization assays were used to assess the effects of substitutions on FaRel2 toxicity. Serial dilutions of *E. coli* strains were plated on solid LB medium and scored after 16 h at 37°C uninduced (grey) and under induction conditions (blue). (*e*) Coomassie-stained SDS–PAGE of purified ATfaRel2 (lane 2; molecular-weight markers are shown in lane 1). (*f*) Coomassie-stained SDS–PAGE of the ATfaRel2–FaRel2^Y128F^ complex (lane 2) and FaRel2^Y128F^ (lane 3); molecular-weight markers are shown in lane 1. The migration of the band corresponding to ATfaRel2 is highlighted by a solid black triangle and that for FaRel2^Y128F^ by an open triangle. (*g*) Analytical SEC of ATfaRel2, FaRel2^Y128F^ and the ATfaRel2–FaRel2^Y128F^ complex on a Superdex 75 Increase column in 25 m*M* HEPES, 300 m*M* NaCl, 1 m*M* TCEP, 2 m*M* MgCl_2_. (*h*) ITC titration to monitor the interaction of ATfaRel2 with its 150 bp operator region.

Due to their extreme toxicity, TA toxins are notoriously difficult to produce recombinantly (Sterckx *et al.*, 2015 ▸). This is particularly true of toxSAS such as FaRel2 (Figs. 1 ▸ *a* and 1 ▸ *b*), FaRel and Tas1 (Jimmy *et al.*, 2020 ▸; Ahmad *et al.*, 2019 ▸). A common strategy to overcome this challenge is the use of catalytically impaired versions of the toxins that have limited enzymatic activity but still retain structural integrity (Garcia-Pino *et al.*, 2016 ▸; Garcia-Rodriguez *et al.*, 2021 ▸; Jurėnas *et al.*, 2019 ▸; Talavera *et al.*, 2018 ▸). The substitution of the conserved tyrosine of the G-loop of SYNTH and toxSYNTH domains (*i.e.* the loop involved in the coordination of pyrophosphate acceptors; Steinchen *et al.*, 2018 ▸) by alanine residues leads to complete inactivation of the toxSAS CapRel and FaRel2 as well as long RSHs (Kurata *et al.*, 2021 ▸; Tamman *et al.*, 2020 ▸; Zhang *et al.*, 2022 ▸). This is likely due to the loss of coordination of the purine base of the acceptor nucleotide. Therefore, to study the toxin FaRel2 we decided to use a more conservative substitution and mutated the aforementioned tyrosine (residue 128) to phenylalanine (FaRel2^Y128F^). We hypothesized that with this substitution, FaRel2^Y128F^ would become catalytically impaired but would still be able to bind uncharged tRNA. Indeed, *in vivo* toxicity neutralization assays show that while FaRel2^Y128F^ remains toxic to *E. coli*, it is dramatically less potent than wild-type FaRel2, and its toxicity is counteracted by the co-expression of the ATfaRel2 antitoxin (Figs. 1 ▸ *c* and 1 ▸ *d*). Thus, this strategy provided us with a system to produce biologically active FaRel2^Y128F^ in sufficient amounts for structural biology studies that could be used to investigate both toxin neutralization and substrate binding.

ATfaRel2 and FaRel2^Y128F^ were both expressed in *E. coli* BL21(DE3) cells using pET-24d and pET-28b expression vectors. Both proteins have a His_6_ tag at the N-terminus followed by a protease cleavage site (Figs. 1 ▸ *e* and 1 ▸ *f*). For ATfaRel2, the cleavage site is specific for TEV protease, while FaRel2^Y128F^ has a SUMO tag specific to UlpI (His_6_-SUMO-FaRel2^Y128F^). In both cases, a two-step purification process was employed. It includes an initial step of affinity chromatography using a Ni–NTA column followed by a second step of size-exclusion chromatography (SEC). Collectively, these steps rendered homogeneous samples. His_6_-SUMO-Farel2^Y128F^ was further subjected to removal of the 6×His-SUMO tag by incubation with UlpI, providing tagless FaRel2^Y128F^. The ATfaRel2–FaRel2^Y128F^ complex was obtained by mixing both proteins followed by SEC to separate the complex from the excess of antitoxin and was confirmed by SDS–PAGE. All preparations were highly pure as judged by SDS–PAGE (Figs. 1 ▸ *e* and 1 ▸ *f*).

### 3.2. Characterization and crystallization of ATfaRel2

We used analytical SEC to determine the oligomeric state of ATfaRel2 (Fig. 1 ▸ *g*). ATfaRel2 eluted at 14.2 ml, which corresponds to an estimated molecular weight of 11.5 kDa consistent with a monomeric species in solution.

In most type II TA operons, transcription is autoregulated via the direct binding of the antitoxin to an operator region in the operon promoter (Hayes & Kędzierska, 2014 ▸). To assess whether ATfaRel2 could interact with its own promoter, we monitored the potential interaction of the antitoxin with a 150 bp DNA fragment derived from the upstream region of the operon by isothermal titration calorimetry (ITC). Analysis of the binding isotherm confirmed the interaction of ATfaRel2 with DNA with an affinity of 484 n*M* (Fig. 1 ▸ *h*).

**Figure 2:**
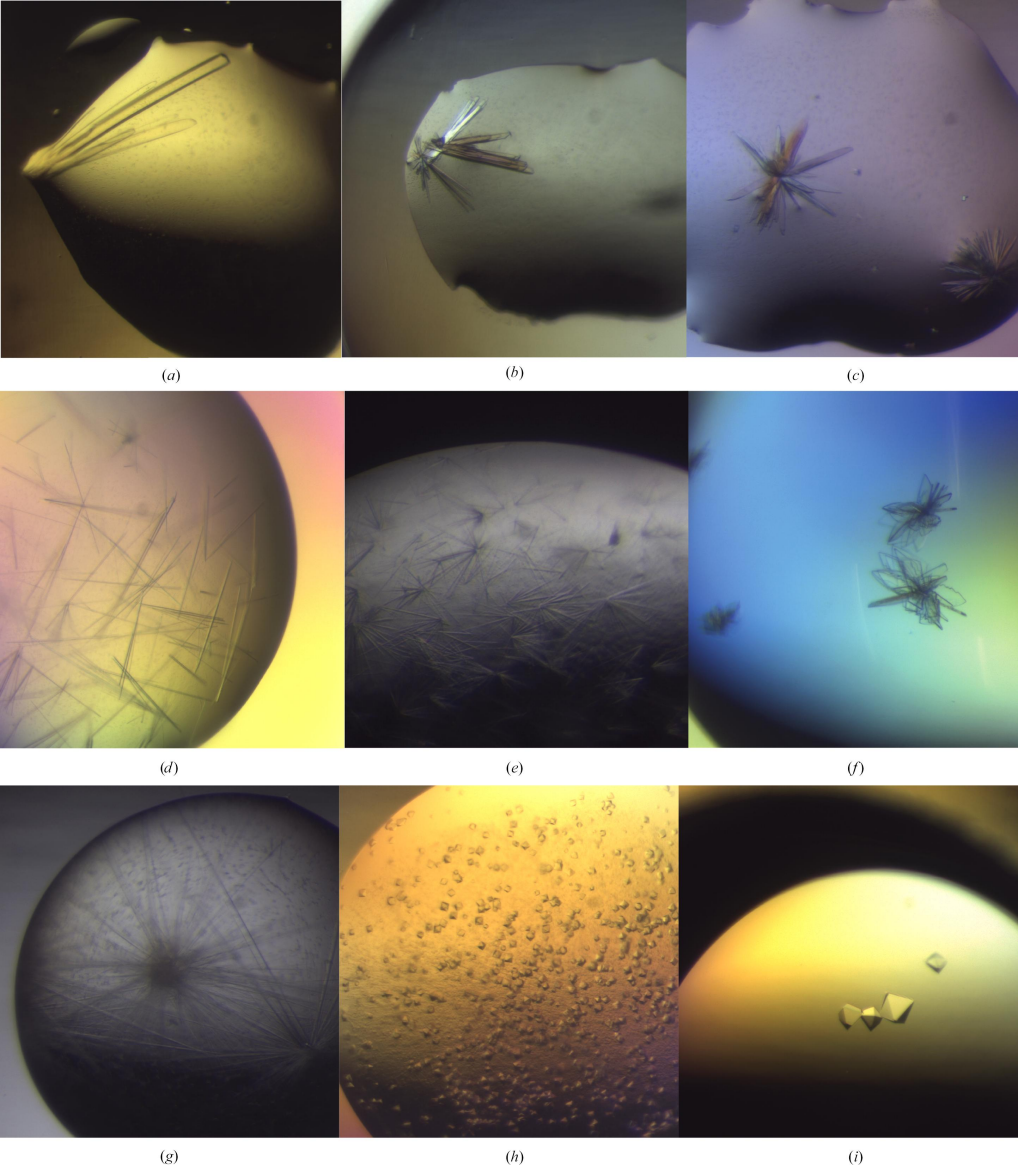
Representative crystals of ATfaRel2 (*a*, *b*, *c*), ATfaRel2 in complex with FaRel2^Y128F^, space groups *P*2_1_2_1_2_1_ (*d*) and *F*4_1_32 (*e*, *f*), and the FaRel2^Y128F^–APCPP complex (*g*, *h*, *i*).

To screen for crystallization conditions for ATfaRel2, the protein was concentrated to 12 mg ml^−1^ in 25 m*M* HEPES pH 7.6, 200 m*M* NaCl, 2 m*M* MgCl_2_, 1 m*M* TCEP. Crystallization hits were observed in several conditions from the LMB screen (Molecular Dimensions; Table 2 ▸). These crystals were directly harvested, soaked in a suitable cryoprotectant solution and vitrified in liquid nitrogen prior to data collection (Table 2 ▸). The crystals grown in 15% PEG 2000 MME and 0.1 *M* bis-Tris propane pH 6.9 (Figs. 2 ▸ *a*–2 ▸ *c*) diffracted to 1.2 Å resolution (Tables 2 ▸ and 4 ▸). The data collected on the PROXIMA-1 beamline of the SOLEIL synchrotron were indexed as orthorhombic. The systematic absences indicated space group *P*2_1_2_1_2, with unit-cell parameters *a* = 53.2, *b* = 34.2, *c* = 37.6 Å. Matthews calculation (Kantardjieff & Rupp, 2003 ▸; Matthews, 1968 ▸) suggested that there is only one molecule in the asymmetric unit, with a low solvent content of 17.4%, *V*_M_ = 1.49 Å^3^ Da^−1^ and probability 1, in agreement with the oligomeric state determined by SEC.

**Figure 3:**
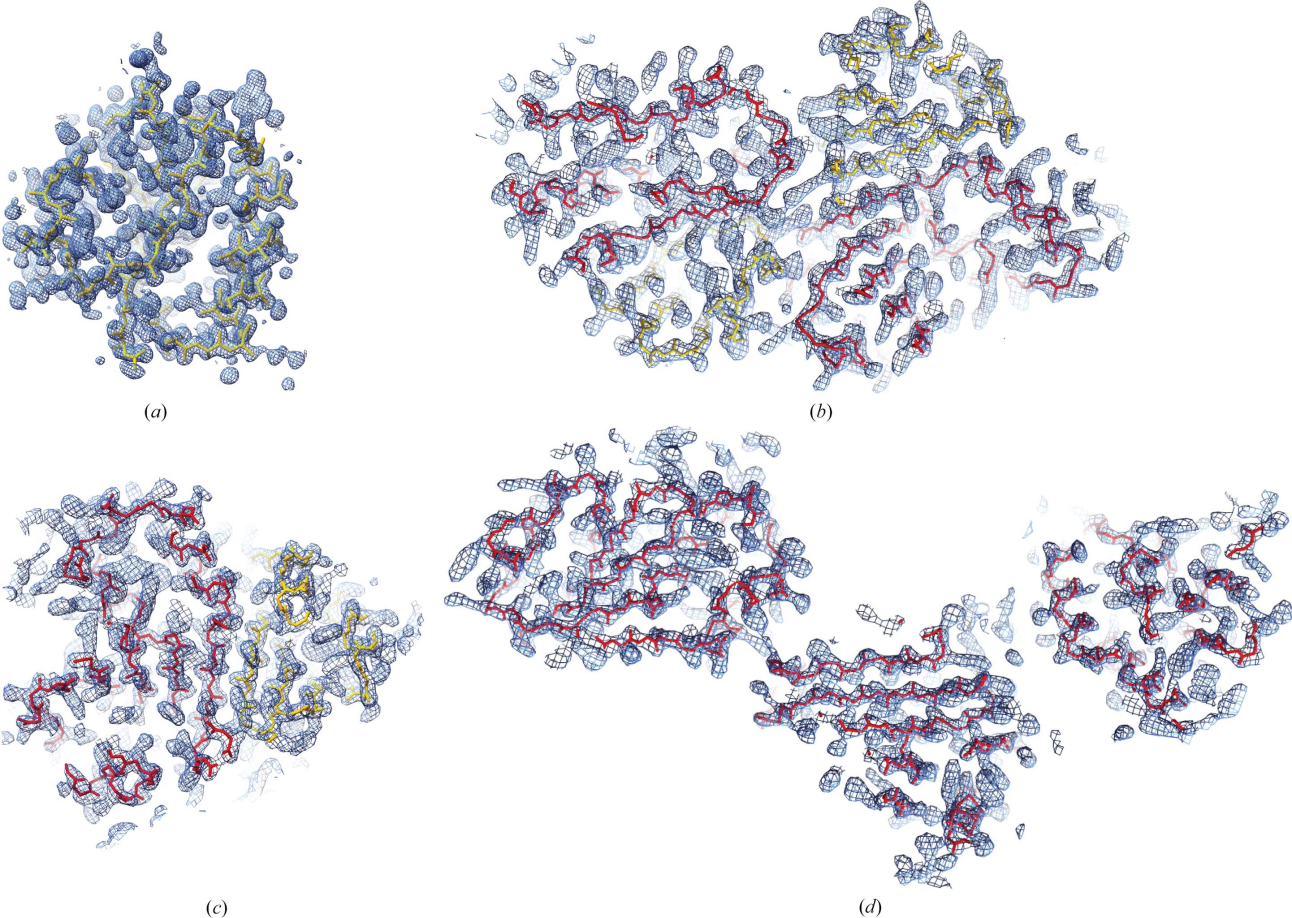
Initial electron-density maps obtained after phasing of ATfaRel2 (*a*), the ATfaRel2–FaRel2^Y128F^ complex in space groups *P*2_1_2_1_2_1_ (*b*) and *F*4_1_32 (*c*), and the FaRel2^Y128F^–APCPP complex (*d*).

A *BLAST* search against the Protein Data Bank (PDB) did not return relevant hits that could be used as search models for molecular replacement. Given the high resolution of the diffraction data and the relatively small size of the protein, we use *ARCIMBOLDO_LITE* (Sammito *et al.*, 2015 ▸) to directly obtain the initial phases. *ARCIMBOLDO_LITE* performs *ab initio* phasing by placing and evaluating single polyalanine α-helices with *Phaser* (McCoy *et al.*, 2007 ▸) and performing density modification and model extension with *SHELXE* (Thorn, 2017 ▸; Usón & Sheldrick, 2018 ▸). The initial search was performed using the default parameters of the program, searching for three generic α-helices each of 16 residues. After density modification, the final solution contained 75 of 99 residues traced into different stretches of alanine peptides with a final *Phaser* *Z*-score of 9.7, which indicates a definite solution (Fig. 3 ▸ *a*).

### 3.3. Characterization and crystallization of the ATfaRel2–FaRel2^Y128F^ complex

To reconstitute the toxin–antitoxin complex *in vitro*, FaRel2^Y128F^ and ATfaRel2 were mixed in a 1:1.2 molar ratio and injected onto an analytical SEC column to separate the complex from excess ATfaRel2. The ATfaRel2–FaRel2^Y128F^ complex eluted at 10.5 ml, which corresponds to an estimated molecular weight of 60 kDa (Fig. 1 ▸ *g*). Therefore, given the molecular weights of the individual proteins, the complex is most likely to be an (ATfaRel2)_2_–(FaRel2^Y128F^)_2_ heterotetramer in solution.

Crystals of the complex grew at 4°C in four different conditions (Figs. 2 ▸ *g*–2 ▸ *i* and Table 2 ▸). Crystals grown in a condition consisting of 35%(*v*/*v*) 1,4-dioxane as the precipiting agent diffracted to 2.1 Å resolution (Table 4 ▸), while crystals that grew in 1 *M* potassium phosphate monobasic, 3%(*v*/*v*) 2-propanol, 0.1 *M* sodium cacodylate pH 6.5 diffracted to 1.9 Å resolution (Table 4 ▸). For the former, diffraction data were collected at DLS. The data were indexed in space group *P*2_1_2_1_2_1_, with unit-cell parameters *a* = 51.7, *b* = 106.6, *c* = 135.1 Å. Matthews calculation strongly supported the presence of an (ATfaRel2)_2_–(FaRel2^Y128F^)_2_ heterotetramer in the asymmetric unit (*V*_M_ = 2.8 Å^3^ Da^−1^, with 52% solvent content and probability 0.72). The crystals from the second condition belonged to the cubic space group *F*4_1_32, with unit-cell parameters *a* = *b* = *c* = 227.1 Å. In this case Matthews analysis could not clearly discern whether the asymmetric unit was composed of one or two ATfaRel2–FaRel2^Y128F^ complexes. For one complex in the asymmetric unit *V*_M_ = 3.67 Å^3^ Da^−1^ with 66% solvent content and probability 0.48, while for two complexes V_M_ = 1.84 Å^3^ Da^−1^ with 33% solvent content and probability 0.52.

To further investigate the unit-cell content of the cubic crystals, we used the partial solution of ATfaRel2 as a search model for molecular replacement with *Phaser*. The MR search could detect only one ATfaRel2 molecule in the asymmetric unit (TFZ = 28.1, PAK = 0, LLG = 457). On the other hand, for the orthorhombic crystals of the complex preliminary MR analysis confirmed the presence of two molecules in the asymmetric unit (TFZ = 15.5 and LLG = 198). These results suggest that ATfaRel2 and FaRel2 are likely to form a symmetrical heterotetramer that crystallizes in space groups *P*2_1_2_1_2_1_ and *F*4_1_32 (in the latter the full complex is likely to be generated by crystallographic symmetry). Thereafter, we used the partial solution found in space group *F*4_1_32 as input for *MR-Rosetta* (Terwilliger *et al.*, 2012 ▸). The program successfully built 216 full residues plus 18 without side chains of a total of 289 (FaRel2^Y128F^ + ATfaRel2; Figs. 3 ▸ *b* and 3 ▸ *c*).

### 3.4. Characterization and crystallization of FaRel2^Y128F^–APCPP complex

FaRel2 hydrolyses the α- and β-phosphate bond of ATP and transfers the β–γ pyrophosphate to the 3′-CCA end of uncharged tRNAs (Kurata *et al.*, 2021 ▸). To study the inter­action between the toxin and the nucleotide substrate, and to avoid its hydrolysis during the structural experiments, we used adenosine-5′-[(α,β)-methyleno]triphosphate (APCPP), a nonhydrolysable analogue of ATP. The FaRel2^Y128F^–APCPP complex was formed by mixing FaRel2^Y128F^ at 19 mg ml^−1^ (562 µ*M*) with 100 m*M* APCPP and was screened for crystallization conditions. Crystals grew in a condition consisting of 50 m*M* MES pH 5.5 plus 12%(*w*/*v*) PEG 2000 and were tested for diffraction on the PROXIMA-2 beamline at the SOLEIL synchrotron (Figs. 2 ▸ *d*–2 ▸ *f*). The best crystal diffracted to 2.6 Å resolution and belonged to space group *P*2_1_, with unit-cell parameters *a* = 31.5, *b* = 60.5, *c* = 177.2 Å, β = 90.6° (Table 4 ▸). Analysis of the unit-cell content (Matthews coefficient *V*_M_ = 2.28 Å^3^ Da^−1^, with a solvent content of 46% and probability 0.86) suggests the presence of three molecules in the asymmetric unit. Using the partial structure of FaRel2^Y128F^ obtained from the ATfaRel2–FaRel^Y128F^ complex as the search model, *Phaser* found a solution with final TFZ = 34.6, LLG = 1509 and PAK = 0 (Fig. 3 ▸ *d*).

## 4. Conclusions

The structural basis of the neutralization of toxSAS by their cognate antitoxins is poorly understood. In particular, it is unclear how homologous antitoxin domains can neutralize similar toxins *in cis* (in the case of fused toxSAS TAs such as CapRel^SJ46^; Zhang *et al.*, 2022 ▸) and *in trans* (in the case of the majority of toxSAS TAs). To overcome the high toxicity of FaRel2 from *Coprobacillus* sp. D7, we used a catalytically impaired version of the toxin, FaRel2^Y128F^. The production of FaRel2^Y128F^ enabled studies of the neutralization of FaRel2 by the ATfaRel2 antitoxin and of how FaRel2 interacts with ATP. Our crystal structures advance the understanding of the function and regulation of the catalytic activity of toxSAS enzymes.

## Figures and Tables

**Table 1:** Macromolecule-production information

Construct	6×His-TEV-ATfaRel2	His-SUMO-FaRel2^Y128F^
Source organism	*Coprobacillus* sp. D7	*Coprobacillus* sp. D7
DNA source	Synthetic	Synthetic
Cloning vector	pET-24d	pET-28b
Expression vector	pET-24d	pET-28b
Expression host	*E. coli* BL21 (DE3)	*E. coli* BL21 (DE3)
Tag	6×His + TEV cleavage site: MKHHHHHHPMSDYDIPTTENLYFQG	6×His + SUMO cleavage site: MGSSHHHHHHGSDSEVNQEAKPEVKPEVKPETHINLKVSDGSSEIFFKIKKTTPLRRLMEAFAKRQGKEMDSLRFLYDGIRIQADQTPEDLDMEDNDIIEAHREQIGG
Complete amino-acid sequence of the construct produced	MKHHHHHHPMSDYDIPTTENLYFQGAMCYIIAKRFKKSGCVALKAKRGKELADFATDLQKKLGYDIQIVAITRPTAYGEYEPYKFVNSFEEFSIEASRL	MGSSHHHHHHGSDSEVNQEAKPEVKPEVKPETHINLKVSDGSSEIFFKIKKTTPLRRLMEAFAKRQGKEMDSLRFLYDGIRIQADQTPEDLDMEDNDIIEAHREQIGGASMYILDKIGLNIEILESLSYESKLGMSFKRTLSHFNKEEVLKEIELINNWYFSLEIIDDLPLDSRIKSVSSAKMKFERYYPNATYNRVFNDILGFRVICKSYDEVLELEKEDKIRVVDMSRGKSNDDGFRGIHVYYQRDNHHYPIEIQFNTYYDRQLNDWLHDKFYKRGYDSSCGQLLRKYYENGKIKSAEELEEVLEDVLYHCKKI

**Table 2:** Crystallization conditions

Enzyme	Protein solution	Reservoir solution	Temperature (K)	Cryoprotectant (collected data sets)
(HisTEV)ATfaRel2	11.65 mg ml^−1^ in 25 m*M* HEPES pH 7.6, 300 m*M* NaCl, 1 m*M* TCEP, 2 m*M* MgCl_2_	**LMB C10**: 15%(*w*/*v*) PEG 2000 MME, 0.1 *M* bis-Tris propane pH 6.9	**277**	**20% glycerol**
(HisTEV)ATfaRel2	11.65 mg ml^−1^ in 25 m*M* HEPES pH 7.6, 300 m*M* NaCl, 1 m*M* TCEP, 2 m*M* MgCl_2_	LMB F5: 29%(*w*/*v*) PEG 4000, 0.1 *M* sodium citrate pH 6.5, 0.1 *M* magnesium acetate tetrahydrate, 0.1 *M* ammonium sulfate	277	20% glycerol
(HisTEV)ATfaRel2	11.65 mg ml^−1^ in 25 m*M* HEPES pH 7.6, 300 m*M* NaCl, 1 m*M* TCEP, 2 m*M* MgCl_2_	LMB D6: 21%(*w*/*v*) PEG 3350, 0.1 *M* MES 6.0, 0.15 *M* sodium chloride	277	20% glycerol
FaRel2^Y128F^ + APCPP	19 mg ml^−1^ + 100 m*M* APCPP in 25 m*M* HEPES pH 7.6, 1 *M* NaCl, 1 m*M* TCEP, 2 m*M* MgCl_2_	**Helix G9**: 0.05 *M* MES pH 5.5, 12%(*w*/*v*) PEG 2000	**277**	**20% glycerol**
FaRel2^Y128F^ + APCPP	19 mg ml^−1^ + 100 m*M* APCPP in 25 m*M* HEPES pH 7.6, 1 *M* NaCl, 1 m*M* TCEP, 2 m*M* MgCl_2_	Crystal Screen 1&2 E4: 35%(*v*/*v*) 1,4-dioxane	277	20% glycerol
FaRel2^Y128F^ + APCPP	19 mg ml^−1^ + 100 m*M* APCPP in 25 m*M* HEPES pH 7.6, 1 *M* NaCl, 1 m*M* TCEP, 2 m*M* MgCl_2_	Nextal H10: 0.05 *M* magnesium chloride, 0.1 *M* MES pH 6.5, 10%(*v*/*v*) 2-propanol, 5%(*w*/*v*) PEG 4000	293	20% glycerol
FaRel2^Y128F^+ (HisTEV)ATfaRel2	10 mg ml^−1^ in 25 m*M* HEPES pH 7.6, 1 *M* NaCl, 1 m*M* TCEP, 2 m*M* MgCl_2_	Crystal Screen 1&2 E4: 35%(*v*/*v*) 1,4-dioxane	277	20% glycerol
FaRel2^Y128F^+ (HisTEV)ATfaRel2	10 mg ml^−1^ in 25 m*M* HEPES pH 7.6, 1 *M* NaCl, 1 m*M* TCEP, 2 m*M* MgCl_2_	**LMB G2**: 3.5%(*w*/*v*) PEG 6000, 0.1 *M* bis-Tris propane pH 7.1, 0.1 *M* potassium chloride	**277**	**40% MPD**
FaRel2^Y128F^+ (HisTEV)ATfaRel2	10 mg ml^−1^ in 25 m*M* HEPES pH 7.6, 1 *M* NaCl, 1 m*M* TCEP, 2 m*M* MgCl_2_	SG1 A5: 0.2 *M* sodium citrate tribasic dihydrate, 20%(*w*/*v*) PEG 3350	277	

**Table 3:** *farel2* promoter sequences used for ITC

Promoter_forward (5′–3′)	ATATTATTTTGGAATAGAAGAATAAAAAGCAAGAAAAGAGGTATTAAATATATGATAAAAAAATATCTGTAATAAGTTTGATTATCAGCAGTTAAGAGATAAACTATGTGAGGTGAATTATTT**ATG**TATATACTTGATAAAATAGGACTT
Promoter_reverse (5′–3′)	AAGTCCTATTTTATCAAGTATATACATAAATAATTCACCTCACATAGTTTATCTCTTAACTGCTGATAATCAAACTTATTACAGATATTTTTTTATCATATATTTAATACCTCTTTTCTTGCTTTTTATTCTTCTATTCCAAAATAATAT

**Table 4:** Data collection and processing

	ATfaRel2	ATfaRel2–FaRel2^Y128F^	ATfaRel2–FaRel2^Y128F^	FaRel2^Y128F^–APCPP
Diffraction source	SOLEIL	SOLEIL	DLS	SOLEIL
Beamline	PROXIMA-1	PROXIMA-2	I24	PROXIMA-2
Wavelength (Å)	0.9801	1.0000	0.9795	1.0000
Temperature (K)	90	90	90	90
Detector	EIGER	EIGER	EIGER	EIGER
Crystal-to-detector distance (mm)	255.730	171.55	289.30	322.94
Rotation range per image (°)	0.1	0.1	0.1	0.1
Total rotation range (°)	720	360	360	360
Space group	*P*2_1_2_1_2	*F*4_1_32	*P*2_1_2_1_2_1_	*P*2_1_
*a*, *b*, *c* (Å)	53.3, 34.3, 37.6	227.1, 227.1, 227.1	51.7, 106.2, 135.1	31.5, 60.6, 177.2
α, β, γ (°)	90.0, 90.0, 90.0	90.0, 90.0, 90.0	90.0 90.0, 90.0	90.0, 90.6, 90.0
Mosaicity (°)	0.2	0.2	0.2	0.2
Resolution range (Å)	30.52–1.24 (1.37–1.24)	80.3–1.98 (2.05–1.98)	80.4–2.14 (3.36–2.14)	59.10–2.62 (2.83–2.62)
Total No. of reflections	191338 (5867)	2656572 (135051)	1074368 (52414)	220686 (8909)
No. of unique reflections	15171 (759)	33494 (1670)	26913 (1346)	16332 (818)
Completeness (%)	92.6 (46.5)	95.3 (52.1)	94.1 (64.3)	90.9 (40.6)
Mutliplicity	12.6 (7.7)	79.3 (80.9)	39.9 (38.9)	13.5 (10.9)
〈*I*/σ(*I*)〉	13.0 (1.3)	14.6 (1.3)	11.3 (1.5)	7.3 (1.4)
*R*_merge_	0.087 (1.205)	0.591 (6.727)	0.33 (4.77)	0.446 (2.730)
*R*_meas_	0.091 (1.289)	0.594 (6.769)	0.33 (4.8)	0.464 (2.864)
CC_1/2_	0.998 (0.711)	0.997 (0.477)	0.99 (0.66)	0.985 (0.332)
Overall *B* factor from Wilson plot (Å^2^)	17.18	27.51	40.97	67.37
